# Time-varying serum gradient of hepatitis B surface antigen predicts risk of relapses after off-NA therapy

**DOI:** 10.1186/s12876-017-0697-3

**Published:** 2017-12-08

**Authors:** Nai-Hsuan Chien, Yen-Tsung Huang, Chun-Ying Wu, Chi-Yang Chang, Ming-Shiang Wu, Jia-Horng Kao, Lein-Ray Mo, Chi-Ming Tai, Chih-Wen Lin, Tzeng-Huey Yang, Jaw-Town Lin, Yao-Chun Hsu

**Affiliations:** 1 Cathay General Hospital, Taipei, Taiwan; 2 School of Medicine, Fu Jen Catholic University, New Taipei, Taiwan; 3 Institute of Statistical Science, Academia Sinica, Taipei, Taiwan; 4 Division of Gastroenterology, Taichung Veterans General Hospital, Taichung, Taiwan; 5 Faculty of Medicine, School of Medicine, National Yang-Ming University, Taipei, Taiwan; 6 Division of Gastroenterology, Fu-Jen Catholic University Hospital, New Taipei, Taiwan; 7 Department of Internal Medicine, National Taiwan University Hospital, Taipei, Taiwan; 8 Graduate Institute of Clinical Medicine, National Taiwan University, Taipei, Taiwan; 9 Department of Internal Medicine, Tainan Municipal Hospital, Tainan, Taiwan; 10 Division of Gastroenterology, E-Da Hospital/I-Shou University, Kaohsiung, Taiwan; 11 Department of Internal Medicine, Lotung Poh-Ai Hospital, Yilan Country, Taiwan; 12 Sijhih Cathay General Hospital, New Taipei, Taiwan; 13 No.510, Zhongzheng Rd., Xinzhuang Dist, New Taipei City, 24205 Taiwan

**Keywords:** Chronic hepatitis B, Nucleos(t)ide analogs, Hepatitis B surface antigen quantification, Time-dependent Cox proportion hazards model

## Abstract

**Background:**

The serum gradient of hepatitis B surface antigen (HBsAg) varies over time after cessation of nucleos(t)ide analog (NA) treatment in patients with chronic hepatitis B (CHB). The association between the time-varying HBsAg serum gradient and risk of relapse has not been elucidated.

**Methods:**

This multicenter cohort study prospectively enrolled CHB patients who discontinued 3 year-NA treatment. Eligible patients were serologically negative for HBeAg and viral DNA at NA cessation. The participants (*n* = 140) were followed every 3 months through HBsAg quantification. Virological and clinical relapses were defined as viral DNA levels >2000 IU/mL and alanine aminotransferase (ALT) levels >80 U/mL, respectively. The association of time-varying HBsAg levels with relapses was assessed through a time-dependent Cox analysis.

**Results:**

During a median follow-up of 19.9 (interquartile range [IQR], 10.6–25.3) months, virological and clinical relapses occurred in 94 and 49 patients, with a 2-year cumulative incidence of 79.2% (95% confidence interval [CI], 70.9%–86.4%) and 42.9% (95% CI, 34.1%–52.8%), respectively. The serum level of HBsAg was associated with virological (*P* < 0.001) and clinical (*P* = 0.01) relapses in a dose–response manner, with adjusted hazard ratios of 2.10 (95% CI, 1.45–3.04) and 2.32 (95% CI, 1.28–4.21). Among the patients (*n* = 19) whose HBsAg levels ever dropped below 10 IU/mL, only one and three patients subsequently developed clinical and virological relapses.

**Conclusion:**

The serum gradient of HBsAg measured throughout the off-therapy observation is associated with the subsequent occurrence of virological and clinical relapses in CHB patients who discontinue NA treatment.

## Background

Chronic infection with hepatitis B virus (HBV) is the leading cause of liver-related morbidity and mortality worldwide, particularly in Asian countries [1]. Management of patients with chronic hepatitis B (CHB) has reached an era of antiviral therapy, with approved regimens consisting of interferon alpha and nucleos(t)ide analogs (NAs) [2–5]. Through the effective inhibition of viral replication, NAs not only ameliorate viremia and reduce hepatic inflammation, but also may prevent and even reverse liver fibrosis [6–8]. A large body of evidence corroborates the effectiveness of NAs in improving clinical outcomes [9, 10]. However, off-therapy durability after discontinuation of NA treatment is typically unsustainable [11–19].

Because of high off-therapy relapse rates, major international guidelines currently recommend an indefinite prolongation of NA therapy, possibly until loss of hepatitis B surface antigen (HBsAg) with or without appearance of accompanying antibodies [12]. However, this strategy entails life-long treatment for most treated patients [13] and is not affordable in regions with resource-constrained health care systems, where, ironically, CHB is most prevalent [14]. Therefore, it is important to find out some factors to predict the risk of off-therapy relapse. Recently, intense research has been carried out to clarify predictors of off-therapy relapse and identify patients who maintain remission without resuming medication [17–22].

In response to the challenges associated with the safe discontinuation of NAs, we prospectively followed a multicenter cohort who discontinued NAs after a minimum of 3 years on therapy. We observed that the serum gradient of HBsAg observed at the end of treatment (EOT), in addition to serum alanine aminotransferase (ALT) levels and age, stratified the risks of both virological and clinical relapses [20]. After the cessation of NA therapy, the level of HBsAg may change over time, but its clinical implication has not been elucidated. Therefore, we conducted this study to explore the association between relapse risks and time-varying serum gradients of HBsAg measured during an off-therapy follow-up.

## Methods

### Design and setting

This prospective cohort study was conducted in three different regional teaching hospitals (E-Da Hospital, Kaohsiung, Lotung Poh-Ai Hospital, Yilan, and National Taiwan University Hospital Yun-Lin Branch, Yunlin) in Taiwan. Institutional review boards approved the study protocol (EMRP100–049) in all hospitals for patient recruitment and database establishment. Data analysis specifically for this study was also approved (EMRP-104-082). Written informed consent was obtained from all participants prior to their enrollment.

### Study participants

We consecutively screened adult patients with CHB who were going to discontinue NA therapy between July 1, 2011 and April 1, 2015, and we evaluated their eligibility. Patients were included if they had been diagnosed with CHB for at least 6 months prior to NA treatment, continuously received any NA (lamivudine, adefovir, telbivudine, entecavir, or tenofovir) for at least 3 years, were serologically negative for HBeAg, and showed undetectable levels of HBV DNA at the end of NA therapy. Patients were excluded in the presence of coinfection with human immunodeficiency virus or hepatitis C virus, any malignancy, liver cirrhosis, hepatic encephalopathy, variceal hemorrhage, organ transplantation, previous use of interferon alpha for 1 month or longer, and concurrent use of cytotoxic or immunosuppressive medication. The diagnosis of liver cirrhosis was based on pathological proof or clinical criteria that included splenomegaly or esophagogastric varices in addition to typical sonographic features.

Patients had to discontinue NAs because of the national health insurance policy. Details of the reimbursement regulations have been previously reported.^10^ In brief, the therapeutic duration was principally restricted to 3 years among general patients without cirrhosis. Those who experienced HBeAg seroconversion on therapy were entitled to treatment consolidation for an additional 1 year.

### Follow-up after cessation of NAs

Pertinent demographic, biochemical, serological, and virological data were collected at enrollment. After discontinuation of NAs, patients were monitored at a close interval of 3 months. The patients underwent physical checkup and laboratory measurement at each follow-up visit. They also underwent abdominal sonography along with serum alpha-fetoprotein estimation tests every 6 months for the surveillance of liver cancer.

Standardized quantification of serum HBsAg and viral DNA was carried out in the Taipei Pathology Institutes (Taipei, Taiwan). Serum HBsAg levels were measured through an automated immunoassay (Abbott Architect i2000, Abbott Park, IL, USA). Samples with HBsAg levels exceeding the upper limit of automatic detection (250 IU/mL) were manually diluted before quantification. Serum HBV DNA was quantified through a commercialized polymerase chain reaction method (COBAS TaqMan HBV Test, version 2.0, Roche Molecular Systems, Inc., Branchburg, NJ, USA) with a detection range of 20–1.7 × 10^8^ IU/mL.

### Definitions of virological and clinical relapses

Virological relapse was defined as the reappearance of >2000 IU/mL HBV DNA in serum. Clinical relapse was defined as an episode of elevated ALT (>80 IU/mL, >2 times the normal conventional upper limit) and >2000 IU/mL HBV DNA. Patients did not resume antiviral therapy until clinical hepatitis persisted for 3 months or longer, unless a risk of hepatic decompensation (serum bilirubin >2 mg/dL or prothrombin time prolonged >3 s) was observed.

### Data analyses and statistical methods

Continuous and categorical variables were summarized using the median and interquartile range (IQR) and proportion with exact numbers, respectively. The incidence rates of virological and clinical relapses were estimated using the Kaplan–Meier method. In a multivariate-adjusted Cox proportional hazards model for off-therapy relapses, the serum level of HBsAg was a time-varying variable that denoted each measurement after NA cessation. The dose–response relationship for the association between HBsAg levels and off-therapy relapses was illustrated by penalized splines in the Cox model. The results were reported as hazard ratios along with 95% confidence intervals (CIs). Data were analyzed using commercial software (Stata, version 13.0; Stata Corp, College Station, TX, USA). All statistical analyses were two-sided with significance set at *P* < 0.05.

## Results

### Baseline characteristics of the participants

**Fig. 1:**
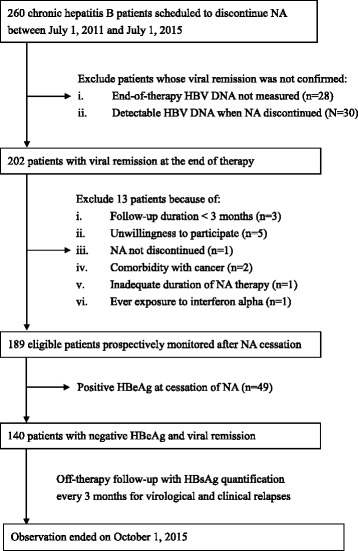
The flowchart for the identification and enrollment of participants

We monitored a total of 140 patients who discontinued NA therapy after a minimum of 3 years (median, 36.6; IQR, 36.4–37.0 months) between July 1, 2011 and April 1, 2015 (Fig. 1). They were followed for a median of 19.9 (IQR, 10.6–25.3) months following the cessation of NA therapy. The mean time interval between initial HBsAg level and last HBsAg level is 19.15 ± 11 months. Table 1 presents a summary of the characteristics of this study population, which was predominantly male (77.9%, *n* = 109) with a median age of 49.1 (IQR, 39.2–57.5) years. Prior to antiviral treatment, 39 patients were initially HBeAg-positive. They were all serologically negative for HBeAg after treatment and had been consolidated for at least 1 year (median, 18.2; IQR, 12.2–25.4 months) following HBeAg loss. The median levels of HBsAg, ALT, and alpha-fetoprotein were 2.79 (IQR, 2.13–3.12) log IU/mL, 22 (IQR, 16.5–34) U/L, and 2.7 (IQR, 1.97–3.41) ng/mL at the end of treatment, respectively.

**Table 1:** Characteristics of the study patients

Characteristics	All patients (*N* = 140)
Male gender, *n* (%)	109 (77.9%)
EOT Age, years	49.1 (39.2, 57.5)
EOT anti-HBe-positive, *n* (%)	131 (93.6%)
EOT HBsAg, log IU/mL	2.79 (2.13, 3.12)
EOT ALT, U/L	22 (16.5, 34)
EOT AFP, ng/ml	2.7 (1.97, 3.41)
Pretreatment HBeAg-positive, *n* (%)	39 (27.9%)
Pretreatment anti-HBe-positive, *n* (%)	100 (71.4%)
Pretreatment viral DNA, log IU/ml	6.21 (4.53, 7.67)
Pretreatment ALT, U/L	154 (95, 451)
On-therapy duration, month	36.6 (36.4, 37.0)
Off-therapy follow-up^a^, month	19.9 (10.6, 25.3)
Patients received Entecavir	125 (89.3%)
Patients received Tenofovir,	9 (6.4%)
Patients received Lamivudine or Telbivudine	6 (4.2%)

Notes. ^a^patients were followed up until reuse of antiviral therapy; *AFP* alpha-fetoprotein, *ALT* Alanine transaminase, *EOT* end-of-therapy, *HBeAg* hepatitis B e antigen, *HBsAg* hepatitis B surface antigen

### Clinical and virological relapses after NA discontinuation

During the off-therapy follow-up, 49 and 94 participants developed clinical and virological relapses, respectively. The cumulative incidence rates of clinical relapses were 28.4% (95% CI, 21.2%–37.5%) and 42.9% (95% CI, 34.1%–52.8%) at 1 and 2 years, respectively, and those of virological relapses were 62.0% (95% CI, 53.5%–70.6%) and 79.2% (95% CI, 70.9%–86.4%) at 1 and 2 years, respectively (Table 2). Seven patients with previous HBeAg positive experienced reoccurrence of HBeAg. Twenty one patients had been treated with NA after relapse. No patient is complicated with hepatic decompensation after secession of NAs therapy in this study.

**Table 2:** Clinical and virological relapses after cessation of nucleos(t)ide analogues in patients with negative HBeAg and undetectable viral DNA at the end of treatment

All (*N* = 140)		
	First year	Second year
Virological relapse	62.0% (95% CI, 53.5–70.6%)	79.2% (95% CI, 70.9–86.4%)
Clinical relapse	28.4% (95% CI, 21.2–37.5%)	42.9% (95% CI, 34.1–52.8%)

### Dose–response relationship of the time-varying HBsAg level and off-therapy relapses

**Fig. 2:**
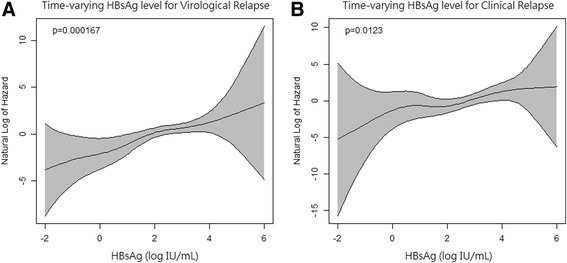
Dose–response relationship for the association of off-therapy HBsAg levels with virological relapse (**a**) and clinical relapse (**b**). The dose–response curves characterized by penalized splines in the Cox model depict a significantly increasing trend; that is, increases in HBsAg levels are associated with increased risks of virological (*P* = 0.00017) and clinical (*P* = 0.012) relapses.Overall, no obvious nonlinearity was observed in the dose–response relationship, supporting the use of a linear term in the Cox model to summarize the hazard ratio for one log increase of HBsAg level

The penalized splines in the univariate Cox model characterized the dose–response curves between increments of HBsAg and risks of relapse (Fig. 2). An increase in the serum level of HBsAg following NA cessation was significantly correlated with a higher risk of subsequent virological (*P* = 0.00017) and clinical (*P* = 0.012) relapses. Moreover, no obvious nonlinearity was observed in the dose–response relationship, supporting the use of a linear term in the Cox model to summarize the hazard ratio for one log increase in the HBsAg level.

### Multivariate-adjusted analyses for the association between time-varying HBsAg levels and off-therapy relapses

The association between time-varying serum levels of HBsAg and off-therapy relapses was further examined through the multivariate-adjusted Cox proportional hazard analysis, which revealed that HBsAg levels, EOT age, and EOT ALT were significant predictors of both clinical and virological relapses. For virological relapse, the serum level of HBsAg, EOT age, and EOT ALT were associated with adjusted hazard ratios of 2.10 per log IU/mL (95% CI, 1.45–3.04), 1.04 per year (95% CI, 1.02–1.06), and 1.02 per U/L (95% CI, 1.01–1.02), respectively (Table 3). Regarding clinical relapse, the serum level of HBsAg, EOT age, and EOT ALT were associated with adjusted hazard ratios of 2.32 per log IU/mL (95% CI, 1.28–4.21), 1.03 per year (95% CI, 1.00–1.06), and 1.03 per U/L (95% CI, 1.02–1.05), respectively (Table 4). Furthermore, the association of HBsAg levels with outcomes did not vary with time (*P* = 0.19 for virological and 0.71 for clinical relapses), thus satisfying the proportional hazard assumption for the model.

**Table 3:** Multivariate Cox porportional hazard model for virological relapse with off-therapy HBsAg level as a time-varying variable

Variables	Adjusted HR	95% CI	*P*
Time-varying HBsAg level, log IU/mL	2.10	1.45, 3.04	<0.0001
Male sex	1.33	0.58, 3.05	0.49
EOT age, year	1.04	1.02, 1.06	0.0005
EOT ALT, U/L	1.02	1.01, 1.02	<0.0001
EOT AFP, ng/mL	1.06	0.96, 1.16	0.24
EOT anti-HBe-seropositive	0.46	0.16, 1.29	0.14
Pretreatment viral DNA, log IU/mL	1.06	0.90, 1.25	0.52
Pretreatment HBeAg-positive	2.58	0.53, 12.50	0.24
Pretreatment anti-HBe-positive	4.02	0.88, 18.3	0.07

Notes. *AFP* alpha-fetoprotein, *ALT* Alanine transaminase, *CI* confidence interval, *EOT* end-of-therapy, *HBeAg* hepatitis B e antigen, *HBsAg* hepatitis B surface antigen, *HR* hazard ratio

**Table 4:** Multivariate Cox porportional hazard model for clinical relapse with off-therapy HBsAg level as a time-varying variable

Variables	Adjusted HR	95% CI	*P*
Time-varying HBsAg level, log IU/mL	2.32	1.28, 4.21	0.005
Male sex	1.05	0.33, 3.29	0.94
EOT age, year	1.03	1.00, 1.06	0.045
EOT ALT, U/L	1.03	1.02, 1.05	<0.0001
EOT AFP, ng/mL	0.88	0.67, 1.15	0.34
EOT anti-HBe-seropositive	1.27	0.14, 11.60	0.84
Pretreatment viral DNA, log IU/mL	1.03	0.79, 1.36	0.81
Pretreatment HBeAg-positive	0.46	0.66, 3.21	0.43
Pretreatment anti-HBe-positive	0.47	0.70, 3.20	0.44

Notes. *AFP* alpha-fetoprotein, *ALT* Alanine transaminase, *CI* confidence interval, *EOT* end-of-therapy, *HBeAg* hepatitis B e antigen, *HBsAg* hepatitis B surface antigen, *HR* hazard ratio

The predictive value of low HBsAg was demonstrated by 19 patients whose respective HBsAg levels ever dropped below 10 IU/mL during the off-therapy follow-up. Among them, only one and three patients subsequently developed clinical and virological relapses, respectively (both *P* < 0.0001), compared with those whose HBsAg levels never fell below10 IU/mL.

## Discussion

In our recent study, we demonstrated that the serum gradient of HBsAg measured at the end of treatment, as well as ALT levels and age, stratified the risks of both virological and clinical relapses [20]. The current study further extends the predictive value of HBsAg measurement beyond the time point at NA cessation. We demonstrated that the off-therapy HBsAg level as a time-varying predictor was associated with both clinical and virological relapses in EOT HBeAg-negative patients with CHB. Eextremely low incidence rates of clinical relapse were observed in those whose HBsAg levels fell below 10 IU/mL, indicating the clinical application of HBsAg surveillance in patients attempting NA discontinuation. Therefore, monitoring serum levels of HBsAg may guide the selection of patients who can maintain sustained viral suppression.

Inactive carriers have been found to have low HBsAg levels [23–25], with HBsAg levels <1000 U/mL having an 87.9% positive predictive value and 96.7% negative predictive value for patients with HBV DNA <2000 IU/mL. A recent study [21] in Northern Taiwan involving 117 patients receiving entecavir treatment reported that serum HBV DNA levels at 3 and 6 months off-therapy were associated with clinical relapse, and the HBsAg level at 6 months off-therapy had a weak association with clinical relapse. Furthermore, 13.6% of the patients had sustained virological response (undetectable HBV DNA levels at 12 months off -therapy). No clinical relapse was observed during a mean 24.8-month follow-up, suggesting that patients without HBsAg clearance could have sustained clinical and viral remission. However, EOT HBsAg was the only factor associated with sustained virological response (*P* = 0.009). Another study involving 252 patients in southern Taiwan [26] also showed that the combination of age (<55 years) and EOT HBsAg levels (<150 IU/mL) was associated with a low rate of virologic relapse. The decline level of HBsAg during treatment was higher in patients with HBsAg clearance than in those without HBsAg clearance. However, both studies mainly relied on EOT HBsAg levels to predict outcomes, and the association of HBsAg kinetics and relapse risk was not discussed. Our study is the first to discuss HBsAg as a time-varying factor for durable remission.

Although patients subjected to NA treatment had a favorable response with regard to HBV DNA levels, the observed HBsAg decline in such patients was lower than in those treated with interferon. This is probably due to the mechanism of NA treatment. NA treatment affects the reverse transcription of pregenomic RNA, but it does not affect covalently closed circular DNA and subgenomic RNA, which have translational activities associated with HBsAg levels [27]. Moreover, serum HBsAg levels reflect active intrahepatic covalently closed circular DNA and have additional value as markers of on-treatment efficacy [28]. However, the NA potentially restores immune responses [29], which possibly results in HBsAg decline. This hypothesis is supported by data that HBsAg decline was strong in patients with higher ALT at the baseline [30] or higher levels of interferon gamma-induced protein 10 at the baseline, which indicates a reasonable level of immune response in such patients. Interferon gamma-induced protein 10 is a chemokine, which is a response to interferon gamma and may reflect the innate immune response against HBV [31]. Our data also show that the serum gradient of HBsAg was associated with the subsequent occurrence of virological and clinical relapses. Especially, the patients, whose HBsAg levels below 10 IU/mL not only at the end of therapy but along the off-therapy follow-up, had very low risk of relapses. Therefore, HBsAg kinetics may be a useful means to monitor NA treatment.

In the current strategies of NA-off follow-up, the HBV DNA and ALT would be monitored routinely and closely. However, elevation of ALT is usually a late event preceded by viral rebound and cannot be regarded as an early predictor. Following abrupt resurgence of viremia, severe acute exacerbation could rapidly ensue and incur the risk of liver failure. Besides, measurement of viral DNA remains costly. Our study showed the HBsAg kinetics has a linear association with relapse risks. Therefore, HBsAg level monitoring, every three months for instance, provides a chance to identify those who may not need frequent viral DNA measurement, and implicates a safe follow-up strategy with less expense.

This study has several strengths. First, we monitored HBsAg levels during the entire follow-up instead of at a single time point. Second, this research was conducted in a real-world setting that reflects “in-field” practice. For external validity, patients were recruited from multiple sites and treatment was not restricted to a single NA. Third, a minimum of 3-year NA therapy enabled most patients to obtain remission status before discontinuing NAs. Finally, stringent monitoring with an interval of 3 months enabled us to observe both virological and clinical relapses closely.

Several limitations were noted in this study. First, because this study prospectively started after NA discontinuation, blood samples prior to the baseline were unavailable. Therefore, we could not ascertain when HBV DNA became undetectable during the treatment by using a standardized protocol. We acknowledge that this is a major limitation because the length of consolidation following remission of viremia has been shown to influence the risk of relapse [14]. Nonetheless, this limitation is unlikely to bias our finding regarding the dose–response association of time-varying HBsAg with off-therapy relapses. Third, we could not quantify pretreatment HBsAg levels exceeding 250 IU/mL and were thus unable to calculate the exact decline of HBsAg during therapy. Intriguingly, a recent small study in Spain suggested that an on-therapy HBsAg decline of more than 5000 IU/mL might indicate durable remission [32]. Finally, because our study exclusively recruited Asian patients whose viral genotype was either type B or C [33], extrapolation to the Western population requires validation.

## Conclusions

In conclusion, this study reveals a dose–response association between time-varying HBsAg and off-therapy relapse following the cessation of NA therapy in patients with CHB. By reporting a negligible risk of subsequent relapse in patients whose HBsAg dropped to a considerably low level (<10 IU/mL), we demonstrate the potential utility of monitoring HBsAg levels in patients with CHB who discontinue NA treatment. These findings may not only implicate a safe strategy to monitor patients who attempt NA cessation but also stimulate further research to elucidate the clinical relevance of HBsAg quantification.

## Abbreviations

ALT: Alanine transaminase
CHB: Chronic hepatitis B
CI: Confidence interval
EOT: End of therapy
HBeAg: Hepatitis B envelope antigen
HBsAg: Hepatitis B surface antigen
HBV: Hepatitis B virus
IQR: Interquartile range
NA: Nucleos(t)ide analog
